# *bicoid* RNA localization requires the *trans*-Golgi network

**DOI:** 10.1186/s41065-019-0106-8

**Published:** 2019-09-10

**Authors:** Xiaoli Cai, Khalid Fahmy, Stefan Baumgartner

**Affiliations:** 1 Department of Experimental Medical Sciences, Lund University, BMC D10, S-22184 Lund, Sweden; 2 Present Address: Department of Genetics, Ain Shams University, Cairo, Egypt; 3 Department of Biology, University of Konstanz, D-78457 Constance, Germany

## Abstract

**Background:**

The formation of the *bicoid* (*bcd*) mRNA gradient is a crucial step for Bcd protein gradient formation in *Drosophila*. In the past, a microtubule (MT)-based cortical network had been shown to be indispensable for *bcd* mRNA transport to the posterior.

**Results:**

We report the identification of a MT-binding protein CLASP/Chb as the first component associated with this cortical MT network. Since CLASPs in vertebrates were shown to serve as an acentriolar microtubule organization center (aMTOC) in concert with *trans*-Golgi proteins, we examined the effect of the *Drosophila trans*-Golgins on *bcd* localization and gradient formation. Using a genetic approach, we demonstrate that the *Drosophila trans*-Golgins *dGCC88*, *dGolgin97* and *dGCC185* indeed affect *bcd* mRNA localization during oocyte development. Consequently, the *bcd* mRNA is already mislocalized before the egg is fertilized. The expression domains of genes downstream of the hierarchy of *bcd*, e.g. of the gap gene *empty spiracles* or of the pair-rule gene *even-skipped* are changed, indicating an altered segmental anlagen, due to a faulty *bcd* gradient. Thus, at the end of embryogenesis, *trans*-Golgin mutants show *bcd*-like cuticle phenotypes.

**Conclusions:**

Our data provides evidence that the Golgi as a cellular member of the secretory pathway exerts control on *bcd* localization which indicates that *bcd* gradient formation is probably more intricate than previously presumed.

**Electronic supplementary material:**

The online version of this article (10.1186/s41065-019-0106-8) contains supplementary material, which is available to authorized users.

## Introduction

In *Drosophila*, the Bicoid (Bcd) concentration gradient along the cortex provides essential positional information on the development of the anterior-posterior axis where it functions as a morphogen. Bcd as a maternal factor is at the top of the hierarchy of segmentation genes that controls via its gradient, the expression of the gap genes which are expressed in broader domains. The gap genes in turn control genes of the next level of the hierarchy, the pair-rule genes. Members of this class are usually expressed in 7 stripes and provide a double-segment identity. The pair-rule genes control the segmentation genes which then provide cues in every single segment. Once the identity of the segments is determined, cells in every segment are specified by the homeotic genes which ensures that these cells do not lose their identity during embryogenesis.

In the past, to explain the occurrence of the morphogen gradient, the hypothesis of a diffusion-driven model (the SDD model, synthesis, diffusion and uniform degradation; [1]) resulting in Bcd gradient formation was widely accepted, securing this concept in the literature for more than two decades. However, in order to establish a stable gradient within 90 min, a diffusion coefficient (D) of Bcd larger than 2 μm^2^/s was calculated to be required for this process [2]. With the advancement of techniques including fluorescence correlation spectroscopy (FCS) and fluorescence recovery after photobleaching (FRAP), the diffusion coefficient could be measured more precisely. Direct and indirect measurements of the Bcd diffusion coefficient were all consistent with D ~ 0.3 μm^2^/s [2], roughly two orders of magnitude lower than expected and thus inconsistent with a simple diffusion model. Several possible scenarios were proposed by [2, 3] to explain the more rapid movement of the Bcd protein. Firstly, Bcd could move faster in the cytoplasm than along the cortex, where the diffusion was measured; secondly, the diffusion rate could change with time, faster during the first hour after fertilization, then slower at later stages, when the coefficient was measured. The third proposal involves active Bcd transport rather than simple diffusion. Since 2007, further progress was made in the accuracy of measuring diffusion constants, and reports claimed higher diffusion rates [4–6]. These were proposed to be sufficiently high enough to explain the SDD model. In contrast, a recent report showed that Bcd does not move in a broad front through the egg, as the SDD model predicts [7, 8], rather it moved along the cortex, as did the mRNA. This data, based on simple experiments immediately refuted the SDD model because it showed that the assumptions of the protein diffusing in a broad front were erroneous.

In 2009, an alternative model was reported [9], termed the ARTS model (active RNA transport and synthesis [7–10]. Here, the formation of the Bcd gradient is based on the existence of a mRNA gradient, mediated by active transport of the mRNA along microtubules (MTs) which exhibits the same variability in shape. This constituted a fourth explanation which resolved most of the constraints that were inherent with the SDD model, reviewed by [8]. Quantitative assays of *bcd* mRNA and Bcd-GFP protein in real-time indicated that the graded mRNA movement made an essential contribution to generating the protein gradient [11]. This finding does not imply that the mRNA diffusion would replace protein diffusion, since the diffusion rate of *bcd* mRNA could be much higher than that of the Bcd protein.

Other models of how the *bcd* gradient could be established were described, an example involving nucleocytoplasmic shuttling of the Bcd protein [12]. In this model, the nuclei would serve as traps to slow down diffusion of Bcd. However, since the nuclei are located in the interior (yolk), while Bcd was shown to move to the periphery [7], the location of the two players is by no means overlapping, thus making this model rather circumstantial, if not obsolete.

This calls into question of how the *bcd* mRNA gradient is established within the same short period. In oocytes, substantial evidence exists that MTs are involved in both transportation and localization of the *bcd* mRNA [13, 14]. Not only *bcd*, but also the movement of the particles of the posterior determinant *oskar* (*osk*), a MT-dependent process that is crucial for its localization at the posterior end [15]. Besides, the RNA binding protein Staufen (Stau) shown to mediate MT-dependent transport, is a member of the large *bcd* ribonuclear protein (RNP) during the first 2 h of development [9, 16]. The entire oocyte MT network is disassembled before egg activation, hence, the fertilized embryo must build up a new MT-based transportation machinery from scratch. Recently, a newly-assembled omnidirectional MT network and a motor for *bcd* mRNA transport was detected at the cortex of early staged embryos [10] fulfilling all the criteria for a transportation system that was predicted [9]. To conclude, active *bcd* mRNA transport as the primary step for Bcd protein gradient formation is now widely accepted, and consistent with the observation of subtle Bcd protein movement along the cortex [7, 8].

It should be noted that all MT-arrays that direct axial patterning are disassembled into short and non-oriented MT filaments throughout the last two stages of oogenesis [17–19], which force the fertilized embryo to build up a new MT network. Consistent with the proposed MT-network for mRNA transport detected by [10], the cortical MTs network resides in the anterior half of early nuclear cycle (nc) 1–6 embryos. To shed more light on the nature of the cortical MTs, we extended our analysis on factors affecting the cortical MT network and *bcd* mRNA transport. We found that *trans*-Golgi components affect the formation of the *bcd* mRNA gradient. Our data demonstrates that the process of *bcd* gradient formation is probably far more complex than previously anticipated.

## Results

### Chromosome bows is part of the MT network that forms the *bcd* mRNA gradient

**Fig. 1:**
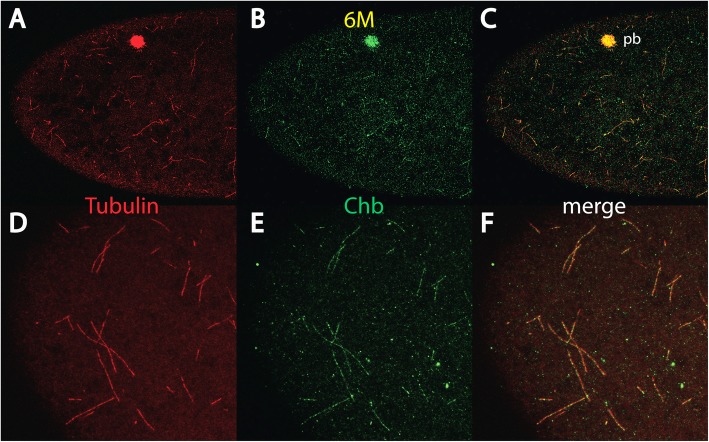
Colocalization of Chb and Tubulin on the anterior MT network. Pictures represent midsagittal confocal planes of embryos oriented with their dorsal side up and anterior to the left. **a**-**c** anterior tip of nc 5 wild-type embryos stained for tyrosinated αTubulin using mab YL_1,2_ (**a**), Chb (**b**) and the merge of (**a** and **b**) in (**c**). **d**-**f** separate confocal section at the anterior tip of the same embryo as in (**a**-**c**) using a high magnification lens, stained for tyrosinated αTubulin using mab YL_1,2_ (**d**), Chb (**e**) and the merge of (**d** and **e**) in (**f**). Note the colocalization of freshly-made MT threads with Chb. Note the strong accumulation of Tubulin and Chb in the polar body (pb) in (**a**-**c**). Chb Chromosome bows. The whole confocal stack was also used to generate a 3D-Video (Additional file 3: Video S1)


To explain the observation of the *bcd* mRNA gradient [9] during early nuclear cycles of *Drosophila* development, a search for a MT-based transportation system was initiated, leading to the discovery of a specific anterior MT network shown to be indispensable for *bcd* mRNA gradient formation [10]. Attempts to define the directionality of the MTs by co-staining the cortical MT threads with minus-end and plus-end markers failed for most markers, possibly because there is no ‘conventional’ microtubule organizing center (MTOC) at the cortex or because the harsh fixation conditions that allowed for the staining of the anterior cortical network were not suitable for antibodies directed against MT-polarity-defining proteins. The only protein that allowed co-localization with the MT threads was Chromosome bows (Chb) [20], formerly called Mast/Orbit/CLASP [21, 22], a protein defining the MT-plus-end (Fig. 1c, f, Additional file 3: Video S1). Chb localization along the MT-threads was not continuous, but appeared rather patchy (Fig. 1, b, c, e, f). The MT-ends were usually free of Chb staining and hence did not allow us to define the directionality of the MT-threads. Interestingly, in vertebrates, Chb was shown to mediate asymmetric nucleation of non-centrosomal MTs at the *trans*-Golgi network, with the help of the *trans*-Golgin marker GCC185 [23, 24]. We reasoned that the specific anterior MT network at the cortex could be nucleated by the *trans*-Golgi network and hence could contribute to build up an acentriolar microtubule organizing center (aMTOC). We therefore sought to investigate the role of *trans*-Golgins in *bcd* localization and gradient formation using genetic approaches that compromise the function of *trans*-Golgins.

**Additional file 3: Video S1** Chb and Tubulin in the anterior MT network. Video of the 3D-reconstruction of the full confocal stack shown as a representative single section in Fig. 1f to reveal the MT network (red) and Chb staining (green) in the anterior half of a nc 5 embryo. (MP4 25986 kb)


### *trans*-Golgins participate in *bcd* localization and gradient formation

The *Drosophila* genome contains four prominent *trans*-Golgin genes identified as *dGCC88*, *dGolgin97* (also called *centrosome’s beautiful sister* (*cbs*)), *dGCC185* and *dGolgin245*, which are structurally well conserved compared to their vertebrate counterparts [25]. We considered if *trans*-Golgins were involved in *bcd* signaling and analyzed the cuticles of *trans*-Golgin mutants in order to identify *bcd*-like phenotypes. Since *dGolgin245* mutants do not show an overt phenotype and are viable [25], the roles of the three remaining *trans*-Golgi proteins were investigated using RNA^i^ fly lines [26, 27].

**Fig. 2:**
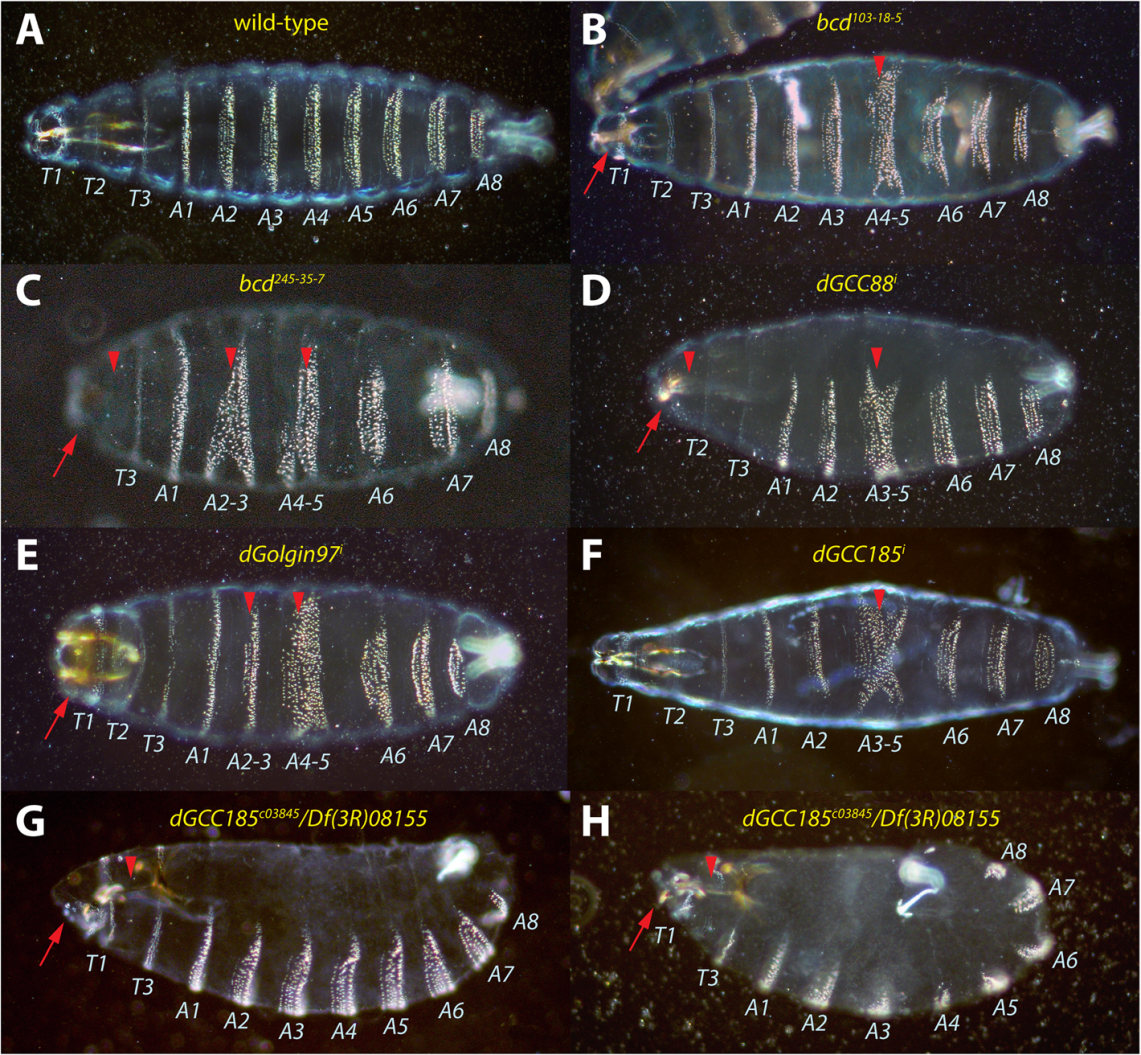
Cuticles pattern of *trans*-Golgin mutants. **a** wild-type larva serving as control. The larval body contains three thoracic (T1-T3) and eight abdominal (A1-A8) segments. Segmental defects are indicated by red arrowheads, head defects as red arrows. **b** embryo from homozygous mothers carrying a weak *bcd*^*103–18-5*^ mutation showing fusion of A4 and A5, as well as subtle head defects. **c** embryo from homozygous mothers carrying a slightly stronger *bcd*^*245–35-7*^ mutation showing pairwise fusions of A4 with A5, A2 with A3, absence of T1 and T2, as well as strong head defects. **d** *dGCC88*^*i*^ embryo showing fusion of A3 to A5, absence of T1, as well as head defects. **e** *dGolgin97*^*i*^ embryo showing fusion of A4 and A5 as well as A2 and A3, but no overt head defect and all thoracic segments present. **f** *dGCC185*^*i*^ embryo showing fusion of A3 to A5, but otherwise mild head defects and all thoracic segments present. **g** *dGCC185*^*c03845*^*/Df(3R)08155* embryo showing lack of T2 and slight defects in germband retraction leading to a round belly and the mouth hooks internalized to the dorsal side*.* **h** *dGCC185*^*c03845*^*/Df(3R)08155* embryo showing lack of T2 and failure to fully retract the germband, as well as head defects and dorsalized mouth hooks, similar to (**g**)

The *GAL4-UAS* system was used [28, 29], as well as a strong maternal *V32* driver in combination with *dGCC88*, *dGolgin97* and *dGCC185* RNA^i^ lines to analyze the cuticle in the knocked-down embryos. The cuticle of two weak alleles of *bcd, bcd*^*103–18-5*^ (Fig. 2b) and *bcd*^*245–35-7*^ (Fig. 2c) [30], respectively, were used as controls. In both *bcd* alleles, abdominal segments 4 and 5 were fused (A4–5) and head defects were observed. *bcd*^*245–35-7*^ proved to be the stronger allele than *bcd*^*103–18-5*^ and in addition showed fusion of A2 and A3 and more pronounced head defects. In addition, a deletion of thoracic segments 1 and 2 (T1, T2) was also observed.

The defects caused by the knock-down of the *trans*-Golgins varied slightly, but were similar to *bcd*^*103–18-5*^ and *bcd*^*245–35-7*^. In *dGCC88*^*i*^ mutants, a fusion of A3-A5 was observed (Fig. 2d), accompanied with head defects and complete deletion of T1. In *dGolgin97*^*i*^ mutants, pairwise fusion of abdominal segments A2–3 and A4–5 were observed (Fig. 2e). Finally, in *dGCC185*^*i*^ mutants, a fusion of A3–5 was observed, but the head region and the thoracic segments did not show any overt phenotype.

Given the importance of *dGCC185* as part of an aMTOC in vertebrates, we sought to establish a ”classical” mutant stock. Since the only available “classical” mutant, *dGCC185*^*c03845*^ harbors a 2nd lethal hit on chromosome 3, we balanced the mutation with a deficiency, *Df(3R)08155* spanning the entire *dGCC185* locus that allowed the establishment of a viable stock, *dGCC185*^*c03845*^*/Df(3R)08155,* with weak embryonic lethality. *dGCC185*^*c03845*^ is a P-element insertion strain causing a deletion the last 67 aa of dGCC185, thereby removing two thirds of the GRIP domain [31] that allows recruitment of dGCC185 to the *trans*-Golgi network. Lethal *dGCC185*^*c03845*^*/Df(3R)08155* embryos displayed a fairly normal cuticle, however, T2 was lacking, the mouth hooks were placed dorsally and the germband was not fully retracted, forcing the embryo into a slightly curved shape (Fig. 2g). In more severe phenotypes (Fig. 2h), the germband showed very little retraction resulting in a complete curved shape. However, this class of mutants revealed similar head and thoracic defects as in the milder mutant phenotype.

**Fig. 3:**
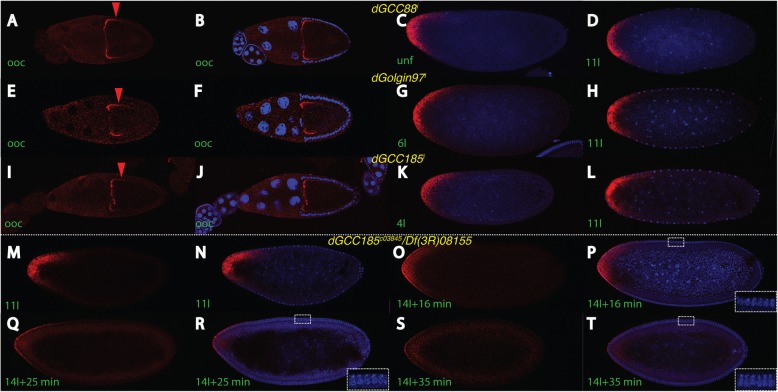
*bcd* mRNA localization in *trans*-Golgin mutants. *bcd* mRNA patterns in oocytes (**a**, **b**, **e**, **f**, **i**, **j**) and embryos (**c**, **d**, **g**, **h**, **k**-**t**) of different *trans*-Golgin mutants, *dGCC88* (**a**-**d**), *dGolgin97* (E-H) and *dGCC185* (I-L), *dGCC185*^*c03845*^*/Df(3R)08155* embryos during different stages at nc 11 and 14 (M-T), as assayed by fluorescent in situ hybridization. **a**, **e**, **i**, **m**, **o**, **q**, **s** *bcd* mRNA pattern as a single signal in red, (**b-d**, **f-h**, **j-l**, **n**, **p**, **r**, **t**) merge of mRNA signal (red) with that of the DAPI channel (blue). Genotypes are indicated in yellow, developmental stages are indicated in green. Nomenclature according to [9, 32, 33]. Enlargements in (P, R, T) denote progression of the nuclear elongation and position of the migrating membrane that allow a precise timing of the onset of nuclear cycle 14, as described [9]

In our next approach, we sought to analyze the effect of *trans*-Golgi genes by monitoring the *bcd* mRNA pattern. We used fluorescent-in-situ hybridization (FISH) to analyze the *bcd* mRNA patterns in knock-down oocytes and embryos. In *dGCC88*^*i*^ oocytes, *bcd* mRNA did not fully localize to the anterior pole (Fig. 3a, b), as in wild-type oocytes (Additional file 1: Figure S1), rather many RNA particles remained localized laterally, demonstrating that the *bcd* mRNA transport was already compromised in the oocyte. This result suggested that Golgi structures must reside in the growing oocyte, consistent with the fact that *dGCC88* is maternally transcribed [20]. Due to the lateral localization of the *bcd* mRNA, an unfertilized egg exhibited a short anterior mRNA gradient (Fig. 3c). Interestingly, this gradient did not change during subsequent nuclear cycles (nc), and a nc 11 embryo still showed the initial shape of the mRNA as in an unfertilized embryo (Fig. 3d), demonstrating that *bcd* mRNA transport in *dGCC88* mutants along the cortex was largely inhibited during early development. This suggested that *dGCC88* plays a role in *bcd* mRNA transport in the embryo, as well.

*dGolgin97*^*i*^ mutant oocytes also showed incomplete *bcd* mRNA transport to the anterior (Fig. 3e, f), similar to *dGCC88* mutants (Fig. 3a, b), and consistent with maternal *dGolgin97* expression [20]*.* As a result, in the early embryo, the mRNA was also distributed in a short anterior gradient (Fig. 3g) that did not change throughout development (Fig. 3h), again similar to the characteristics displayed in *dGCC88* mutants (Fig. 3d).

In *dGCC185*^*i*^ oocytes (Fig. 3i, j), the effect on *bcd* mRNA localization was minimal and the mRNA transport to the anterior end was almost indistinguishable from wild-type (Additional file 1: Figure S1), suggesting that *dGCC185* did not have a strong effect on mRNA localization in oocytes. In embryos (Fig. 3k, l), the effect on mRNA transport was subtle and the gradients appeared normal, suggesting that *dGCC185* did not have a strong effect on mRNA localization in embryos as well.

In early *dGCC185*^*c03845*^*/Df(3R)08155* nc embryos, the localization of the *bcd* mRNA was indistinguishable from that of wild-type embryos (data not shown), as evident from nc 11 embryos (Fig. 3m, n). The only overt phenotype occurred during nc 14 at a time point when the *bcd* mRNA is transported from the basal to the apical side, followed by rapid degradation after 16 min after the onset of nc 14, (nc14 + 16 min; [9]. We observed a substantial delay in *bcd* mRNA degradation in a *dGCC185*^*c03845*^*/Df(3R)*08155 mutant embryo, exemplified by an embryo where no mRNA degradation has yet occurred (nc 14 + 16 min; Fig. 3o, p). In comparison, in an identically-staged wild-type embryo, the mRNA was already degraded and was no longer visible [9]. The mRNA was still visible on the apical side of nc14 + 25 min-old embryos (Fig. 3q, r) and degradation was completed in nc14 + 35 min old embryos only (Fig. 3s, t). This corresponded to about 20 min more longevity compared to wild-type, suggesting that *dGCC185* was either directly involved in basal-apical transport, or that *dGCC185* provided a temporal signal when the basal-apical transport should be initiated. We note that these phenotypes are subtle, most likely due to the fact that the mutation is hypomorphic.

**Fig. 4:**
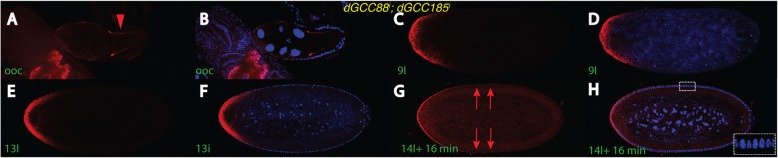
*bcd* mRNA localization in a *dGCC88*^*i*^; *dGCC185*^*i*^ double mutant. *bcd* mRNA patterns in oocytes (**a**, **b**) and embryos (**c**-**h**) of the *dGCC88*^*i*^; *dGCC185*^*i*^ double mutant combination, as assayed by fluorescent in situ hybridization. **a**, **c**, **e**, **g** *bcd* mRNA pattern in red, (**b**, **d**, **f**, **h**) merge of mRNA signal (red) with that of the DAPI channel (blue). The genotype is indicated in yellow. Developmental stages are indicated in green, nomenclature is according to [9, 32, 33]. Note the lateral localization of the *bcd* mRNA in mutant oocytes (red arrowhead). Enlargement in (**h**) denotes progression of the nuclear elongation position of the migrating membrane that allow a precise timing of the stage of the nuclei following onset of nc 14. Red arrows in (**g**) denote mRNA particles up to the middle of the embryo

*trans*-Golgins exert their function in concert with other *trans*-Golgins, linked via the C-terminal coiled-coil region GRIP [34]. Hence, it was presumed that the knock-down of one member may not lead to complete loss-of-function of the whole assembly, rather it would be weakened. We sought to analyze the effect of these proteins in a double-mutant background by recombining two single RNA^i^ lines together to produce a double mutant *dGCC88*^*i*^; *dGCC185*^*i*^ RNA^i^ line. In these double mutants, the mRNA is not transported to the anterior side, rather it stays entirely at the lateral portion (Fig. 4a, b). This data clearly demonstrated an additive effect and consequently a more severe phenotype compared to a single mutation alone (Fig. 3a-b, i-j). As a consequence of this lateral localization in oocytes, transcripts were found in a broad gradient in early embryos (Fig. 4c, d), which extended even more at nc 13 (Fig. 4e, f) and finally peaking during nc 14 (Fig. 4g, h) where transcripts were transported up to the middle of the embryo and thus much farther than in wild-type embryos [9, 10]. Moreover, *bcd* transcripts lingered slightly longer comparable to those of a nc 14 wild-type embryo and were still readily visible apically at nc 14 + 16 min, (Fig. 4g, h). However, they did not persist as long as in seen in the “classical” *dGCC185* mutant (Fig. 3s, t).

### Effect of *trans*-Golgins on *bcd* downstream targets

**Fig. 5:**
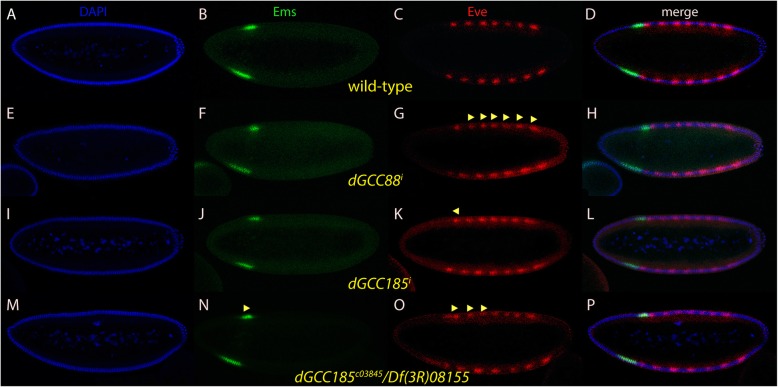
Ems and Eve expression patterns in *trans*-Golgin mutants. Pictures represent midsagittal confocal planes of embryos oriented with their dorsal side up and anterior to the left. **a**-**d** nc 14 wild-type embryo stained for DAPI (**a**), Ems (**b**), Eve (**c**) and the merge of (**a**-**c**) in (**d**). **e**-**h** nc 14 *dGolgin88*^*i*^ embryo stained for DAPI (**e**), Ems (**f**), Eve (**g**) and the merge of (**e**-**g**) in (**h**). **g** shows that Eve stripes 2, 3, 4, 5, 6 and 7 are shifted towards the posterior. (I-L) nc 14 *dGCC185*^i^ *III* embryo stained for DAPI (**i**), Ems (**j**), Eve (**k**) and the merge of (**i**-**k**) in (**l**). **k** shows that Eve stripe 1 is shifted towards the anterior. **m**-**p** nc 14 *dGCC185*^*03845*^*/Df(3R)08155* embryo stained for DAPI (**m**), Ems (**n**), Eve (**o**) and the merge of (**m**-**o**) in (**p**). **o** shows that the Eve stripes 1, 2, 3 are shifted towards the posterior. ◀ showing shift towards the anterior. ▶ showing shift towards the posterior

Since the cuticles of the *trans*-Golgins mutants exhibited various defects related to *bcd*, targets downstream of the segmentation gene hierarchy such as the gap gene *empty spiracles* (*ems*) and the pair-rule gene *even-skipped* (*eve*) were chosen to monitor the activity of *bcd*, based on their altered expression patterns (Fig. 5a-d). We compared the position of the stripes of RNA^i^-mediated mutant embryos along the A-P axis to those of wild-type embryos through t-test analysis (Tables 1, 2; Additional file 2: Figure S2). Interestingly, only *dGCC88* RNA^i^ embryos resulted in a significant difference in the stripe pattern (Fig. 5e-h, Additional file 2: Figure S2). In *dGCC88*^*i*^ embryos, Eve stripes 2 (0.01 < *P* < 0.05), 3 (*P* < 0.01), 4 (*P* < 0.01), 5 (*P* < 0.01), 6 (0.01 < *P* < 0.05), and 7 (*P* < 0.01) shifted towards the posterior significantly (Fig. 5g, arrowheads, Additional file 2: Figure S2). Eve stripe 1 (*P* > 0.05) and Ems (*P* > 0.05) was an exception (Additional file 2: Figure S2). Surprisingly, the other *trans*-Golgi genes, in particular *dGCC185* did not behave as expected and did not show any statistically significant change (Additional file 2: Figure S2). To rule out variations in the expression levels of the transgenes in the RNA^i^ lines, two different lines of *dGCC185*^*i*^ were tested, one with an insertion on the 2nd chromosome, termed II, and another on the 3rd chromosome, termed III. In the *dGCC185*^*i*^ *II* line*,* the shift of patterns of Ems and Eve was not statistically different from that of a wild-type embryo (P > 0.05) (Additional file 2: Figure S2). In the *dGCC185*^*i*^ *III line*, however, although only Eve stripe 1 showed a significant shift (0.01 < *P* < 0.05; Fig. 5k, arrowhead), it was towards the anterior (Additional file 2: Figure S2). Considering the importance of *dGCC185* for the *trans*-Golgi network, it was reasoned that the maternal driver *V32* was too weak a driver to mediate sufficient down-regulation of *dGCC185*. For this reason, a triple maternal driver (*MTD*, the strongest maternal driver available in the stock centers), was used in combination with *dGCC185 III*. Surprisingly, the results did not show a significant difference compared to the *V32* driver (data not shown). However, when embryos from the *dGCC185*^*c03845*^*/Df(3R)08155* mutant combination were analyzed, Ems (0.05 < *P* < 0.01), Eve stripes 1 (*P* < 0.01), 2 (*P* < 0.01) and 3 (0.01 < *P* < 0.05) showed a posterior shift (Fig. 5n, o, arrowheads; Additional file 2: Figure S2).

**Table 1:** Shift of Even-skipped (Eve) stripes in *trans*-Golgi mutants. 0–4 h embryos from wild-type and *trans*-Golgi protein mutants were collected at 29 °C, respectively. Embryos were stained with an antibody against Eve. Eve stripes were measured by ImageJ. Data was analyzed by using Student’s t-test (*p*-value of 0.05) and reported as means ± SE

a	1^st^	2^nd^	3^rd^	4^th^	5^th^	6^th^	7^th^	T (^o^ C) n
wild type	29.30% ± 0.85%	37.56% ± 1.02%	46.35% ± 0.74%	53.83% ± 0.90%	61.19% ± 0.90%	68.69% ± 1.03%	77.09% ± 1.10%	29 16
*dGCC88* ^*i*^	29.39% ± 0.91%	38.44%* ± 0.67%	47.73%** ± 1.10%	55.43%** ± 1.15%	63.17%** ± 1.49%	70.23%** ± 1.67%	78.65%** ± 1.53%	29 10
*dGolgin97* ^*i*^	29.70% ± 0.96%	38.30% ± 0.93%	47.36%** ± 0.99%	54.85%* ± 1.30%	62.24%* ± 1.78%	69.16% ± 1.80%	77.33% ± 1.65%	29 10
*dGCC185* ^*i*^ *(III)*	28.35%* ± 1.06%	36.79% ± 1.20%	45.70% ± 1.18%	53.80% ± 1.24%	61.10% ± 1.57%	67.78% ± 1.64%	76.13% ± 1.57%	29 13
*dGCC185*^*c03845*^/ *Df(3R)08155*	31,44%** ± 1.30%	39,27%** ± 1.30%	47,28%* ± 1.30%	54,42% ± 1.33%	61,70% ± 1.35%	68,57% ± 1.28%	76,89% ± 1.56%	29 17

^a^: Eve stripe number

n: Number of embryos examined

i: RNA^i^

* 0.05 < *P* < 0.01; ** *P* < 0.01

**Table 2:** Shift of Empty spiracles (Ems) band in *trans*-Golgi mutants. 0–4 h embryos from wild-type and *trans*-Golgi protein mutants were collected at 29 °C, respectively. Embryos were stained with an antibody against Ems. The Ems band was measured by ImageJ. Data were analyzed by using Student’s t-test (*p*-value of 0.05) and reported as means ± SE

		T (^o^ C) n
wild type	29.61% ± 1.06%	29 16
*dGCC88* ^*i*^	29.35% ± 1.09%	29 10
*dGolgin97* ^*i*^	30.11% ± 0.96%	29 10
*dGCC185* ^*i*^ *(III)*	28.96% ± 0.94%	29 13
*dGCC185* ^*c03845*^ */ Df(3R)08155*	30,63% ± 1.09%*	29 17

n: Number of embryos examined

i: RNA^i^

* 0.05 < *P* < 0.01

**Fig. 6:**
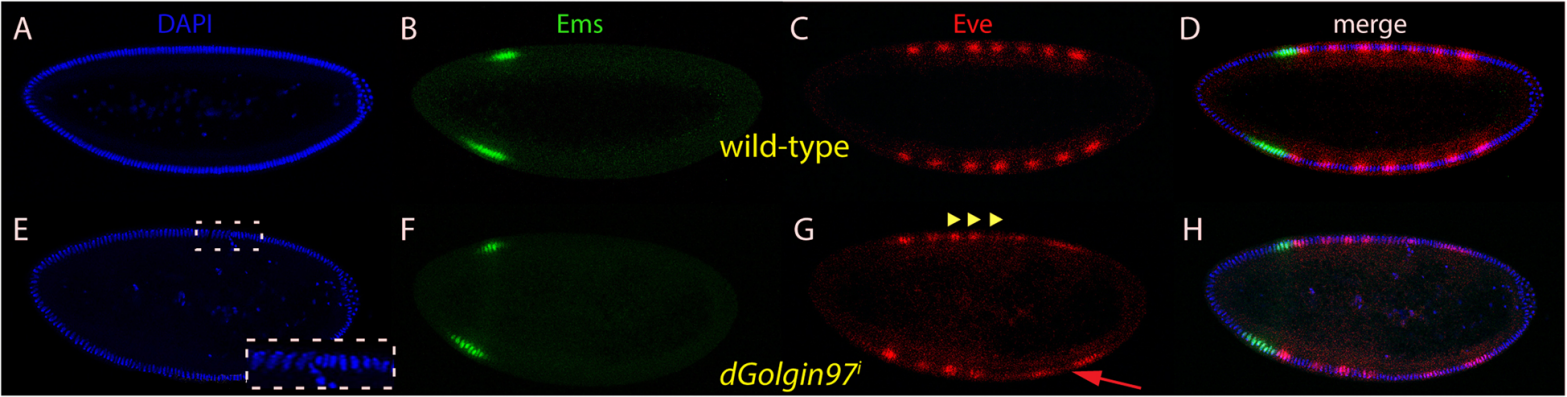
Ems and Eve expression patterns in the *dGolgin97* mutant. Pictures represent midsagittal confocal planes of embryos oriented with their dorsal side up and anterior to the left. **a**-**d** nc 14 wild-type embryo stained for DAPI (**a**), Ems (**b**), Eve (**c**) and the merge of (**a**-**c**) in (**d**). **e**-**h** nc 14 *dGolgin97*^*i*^ embryo stained for DAPI (**e**), Ems (**f**), Eve (**g**) and the merge of (**e**-**f**) in (**h**). (**e**, insert) close up of the area showing different density of nuclei along the dorsal side. **g** shows Eve stripes 3, 4, and 5 shifted towards the posterior. Eve stripes 5, 6 and 7 are delayed at nc 14. ▶ showing shift towards the posterior. Red arrow indicates delay of formation of Eve stripes 5–7

In contrast to the other *trans*-Golgi proteins, *dGolgin97*^*i*^ revealed a mild posteriorward shift of Eve stripes 3 (*P* < 0.01), 4 and 5 (0.01 < *P* < 0.05) (Fig. 6g, arrowheads; Additional file 2: Figure S2). Interestingly, in *dGolgin97* mutant embryos, the nuclei covering the anterior 60% of the embryo were at an advanced stage compared to those of the posterior 40% (Fig. 4e, insert) suggesting that *dGolgin97* affects the maturation of the nuclei during nc 14 in a spatial manner. Consequently, the formation of stripes 5–7 was delayed and the stripe pattern was not resolved yet (Fig. 6g, arrow).

## Discussion

Our study provides evidence that the simple SDD diffusion model proposed more than 3 decades ago by [1] needs to be revised in order to explain the movement of Bcd for gradient formation [8]. Our data demonstrates that the *bcd* mRNA gradient is the template for the Bcd protein gradient, also termed the ARTS model [9, 10].

Our previous analysis revealed that MT threads at the anterior pole is a prerequisite for the ARTS model and *bcd* mRNA transport [10]. Attempts to localize the minus- or plus end-binding proteins on the cortical MT threads failed, possibly because there is no “conventional” microtubule organization center (MTOC) at the cortex, or because the harsh fixation conditions led to denaturation of the antigens and thus prevented the detection using the panel of antibodies that were available. The only protein that showed co-localization with the cortical MTs was Chromosome bows (Chb), also named CLASP/MAST/Orbit [21, 35], a plus end-binding protein, which decorated the MTs in a patchy manner (Fig. 1). Recent studies showed that by using a CLASP-dependent nucleation at the *trans*-Golgi network, asymmetric MTs could be nucleated in a centrosome-independent way, originating from an aMTOC [23].

Considering the role of the *trans*-Golgins as an aMTOC, we reasoned that early cortical Golgi structures could help to initiate and organize cortical MTs (Fig. 1a, d). Little is known about the presence of cortical Golgi structures during early nuclear cycles [36]. The only description of Golgi structures in the early embryonic cortex was published by [37]. This work demonstrated that Golgi structures were not only associated with dividing nuclei in the interior of embryo, but they were also found at the cortex. The same report also described Golgi-like structures at the cortex of growing oocytes, suggesting that these structures are already built up maternally, consistent with our data on the effect on down-regulating *trans*-Golgins in oocytes (Fig. 3). Another report showed the presence of punctate wheat germ agglutinin (WGA)-positive structures to denote *trans*-Golgi structures at the cortex of early nc embryos [38]. A similar punctate cortical staining is indeed observed when staining early nc embryos for the presence of the coatomer Golgi marker β-COP (data not shown). The data demonstrates that Golgi-like structures do exist, at the cortex of early nc embryos, but more research is needed to elucidate origin and nature of these structures.

In 2007, it was reported that members of the ESCRT-II endosomal sorting complex had profound effects on *bcd* mRNA localization in the oocyte [39], similar to the effect seen in the *dGCC88*^*i*^; *dGCC185*^*i*^ double mutant. The key proteins of the ESCRT-II complex, VPS22, VPS25 and VPS36 all showed an effect on *bcd* mRNA localization during oogenesis, while only VPS36 showed physical binding to the 3’UTR of *bcd* [39]. While the mechanisms underlying *bcd* localization in oocytes are clearly different from those proposed to occur with the *trans*-Golgins, it suggests that several elements of the secretory pathway can have an effect on *bcd* localization.

*dGCC185,* as the only member of the 4 *trans*-Golgins reported to be involved in MTs nucleation did not show any significant shift of the Eve stripes. In contrast, *dGCC88* showed the most significant shift of the Eve stripes. As discussed by [23], even under conditions where GCC185 function is compromised and the CLASPs lose their association with the Golgi stacks, it does not affect displaced CLASPs for stabilization of pre-existing MTs seeds and to promote their growth. In fact, there could be other *trans*-Golgins that compensate for the loss of a particular Golgin. For example, the deletion of either GMAP210 or GM130, two *cis*-Golgi resident proteins exhibiting similar phenotypes suggests that the two Golgins function in similar pathways [40, 41]. In particular, the latter, GMAP210 possesses a MT-minus-end binding activity [42], making it another prime candidate for Golgi-mediated MT-binding. As far as nucleation of MTs is concerned, according to our results, dGCC88 would constitute the most important player where most of the *bcd* signal is relayed, as it showed the strongest shift of the Eve stripes (Fig. 5g, Table 1) and changes in the cuticle (Fig. 2d). Thus, to address the question as to the compensation of function between Golgins and the fact that all Golgins bind small G-proteins via their GRIP domains to exert their functions, we subjected two G-proteins, Rab6 and Arl1 (Arf-like1) to the same analysis as the Golgins (data not shown). Rab6 belongs to the Rab family of small G-proteins, and is located to the *trans*-Golgi compartment to recruit Golgins and enable membrane trafficking [43]. Arl1 is the small G-protein Arf-like1 and is also located on the Golgi complex to recruit Golgins [44]. Again, no significant shift of the Eve stripes was seen in *rab6* and *arl1* mutants (data not shown). [45] pointed out that in mammals, it is still a conundrum that Arl1 is able to bind the GRIP domain of GCC185, and questionable whether an interaction with Rab6 is essential for Golgi function. Even if the relationship between G-proteins and *trans*-Golgi proteins is an important one, the interaction may not be exclusive, suggesting that a single Golgin may not carry a specific function, but rather work together with other Golgins at the *trans*-Golgi surface. In a situation where a partial-loss of either *rab6* or *arl1* is achieved, the four *trans*-Golgins would still not lose their association with the Golgi structure completely. This was demonstrated by [44], showing that loss of *arl1* function leads to mislocalization of dGCC88, dGolgin97 and dGolgin245, but not of dGCC185.

The study by [46] identified that a maternal effect comes into play to account for the variability of the developmental time of the embryo. Given the nuclear density differences in *dGolgin97*^*i*^ mutants at 0–60% egg length (Fig. 6e), we hypothesize that apart from shifts of the Eve and Ems stripes in *trans*-Golgins mutants, the variance of the developmental time could be an option for alterations seen in *bcd* signaling as well.

Interestingly, none of the *trans*-Golgin mutations showed 100% identical cuticle defects inherent with the two weak *bcd* alleles (Fig. 2b, c). Always in common was a fusion of A4 to A5, to a lesser extent also between A2 to A3 (Fig. 2d-f) and head defects (Fig. 2d, e). Of the *trans*-Golgins, *dGCC88*^*i*^ embryos showed the most severe cuticle defects, also documented by the strongest variation in the shift of the Eve stripes (Fig. 5, Additional file 2: Figure S2). This suggests distinct requirements for function or assembly of the *trans*-Golgi network, or for localization of the *bcd* transcripts in the oocyte. The fact that none of the mutants showed mRNA transportation activity in the embryo suggests that the RNA^i^ approach compromised all maternal contribution of the *trans*-Golgins and thus prevented any *trans*-Golgin activity in the embryo.

As far as the “classical” *dGCC185* mutant and its particular phenotype is concerned (Fig. 2g, h), only the head defects and absence of T2 were reminiscent of the weak *bcd* phenotype. The P-element insertion leads to a deletion of the last 67 aa leading and thus to a partial deletion of the GRIP domain which confers binding to Arl1 to mediate Golgi recruitment [25]. In this COOH-terminal-truncated dGCC185 protein, the vast majority of the coiled-coil part is still intact and projects, in concert with the other *trans*-Golgins-like tentacles into the cytoplasm. It is therefore not surprising that the cuticle phenotype is rather mild and not fully reminiscent of the other *trans*-Golgin RNA^i^ mutant lines. Moreover, *bcd* mRNA localization in oocytes and embryos was indistinguishable from that of wild-type (Fig. 3m, n, Additional file 1: Figure S1), except for the transcripts being more persistent during nc 14 (Fig. 3o-t). Here, dGCC185 could provide a function for the basal-apical transport of the mRNA, as this one is mediated by MTs. Hence, the truncated dGCC185 protein could lower the activity of this transport and thus delay the degradation of the mRNA on the apical side.

The majority of the *trans*-Golgin lines did not show a significant shift of the gap gene Ems, which could be explained by the results from the study of [47]. Their assay on genome-wide measurement of spatial expression in patterning mutants of *Drosophila* implied that only several key transcription factors would show significant expression pattern changes in *bcd* mutants. This could mean that even though the expression of Ems is *bcd*-dependent, Ems is not a key transcription factor during the earlier stage. Thus, without a sufficient change in the activity of Bcd, Ems will not respond substantially, particularly taking into account that the concentrations of Bcd at the position where Ems is expressed are still rather high. Hence, the position where Ems is expressed is less sensitive to fluctuations in Bcd levels.

## Conclusions

Our data provides evidence that, apart from the ESCRT II system, another element of the secretory pathway, i.e. that of the Golgi system, plays a pivotal role in *bcd* mRNA localization. This calls into question this simplistic model of how the *bcd* gradient is established and may suggest that *bcd* gradient formation might be dependent on far more components than previously anticipated, a notion that should be taken into consideration when working with this paradigm for gradient formation.

## Materials and methods

### *Drosophila* stocks and genetics

Canton-S stock from Bloomington (No. 64349) was used as a control. The maternal *GAL4*-driver line *V32* was obtained from Perrimon lab. All of the *UAS* fly strains were obtained from the Bloomington or Vienna *Drosophila* Stock Centers. A viable stock of *dGCC185*^*c03845*^ *in trans* to *Df(3R)08155* was utilized to generate embryos with a COOH terminal deletion (the last 67 aa) in dGCC185.

Flies were fed with standard fly food (Bloomington recipe) and were maintained at either 25^°^C or 29^°^C, depending on the efficacy of the maternal driver system.

### Embryo fixation for cortical MTs network staining

Embryos in Fig. 1 were fixed at high concentrations of formaldehyde (> 25%), as described in [10].

### Cuticle preparations

Embryos were collected in 24 h. intervals, incubated > 36 h., dechorionated in 50% bleaching solution, fixed in 25% formaldehyde for > 5 h., devitellinized, mounted in Hoyer’s and incubated at 65^°^C for 3–5 days, as described [7].

### Antibody staining and fluorescent in situ hybridization

The working concentration for mab YL_1, 2_ against tyrosinated tubulin (Thermo Fisher Scientific) was at 1:2000. The rabbit polyclonal antibody against Chb was obtained from Claudio Sunkel and was used at 1:500. The monoclonal antibody 2B8 against Eve (DSHB) was used at 1:250. Rabbit-anti-Ems antibodies were obtained from Uwe Walldorf and were used at 1:1000. DAPI for nuclear staining was used at 1:1000 from a 1 mg/ml stock.

The protocol for fluorescent in situ hybridization was adopted from [10], with the exception that RNA probes were tagged with an Alexa Fluor 568 Signal-Amplification Kit (Invitrogen A11066).

### Data analysis

All images were recorded using a Zeiss LSM 710 confocal microscope. Images were post-processed with Adobe Photoshop and Adobe Illustrator. Image J was used to measure the length of the embryos, the distance between the anterior tip, the anterior border of each *eve* stripe and the posterior border of *ems*. All data was analyzed with Analysis of Variance (ANOVA) and two-tailed Student’s t-tests. Data are reported as means ± SE.

## Additional files

Additional file 1: **Figure S1.** *bcd* mRNA expression in wild-type embryos. *bcd* expression pattern in wild-type oocytes, as assayed by fluorescent in situ hybridization. (A) *bcd* mRNA pattern as a single signal in red, (B) merge of the mRNA signal (red) with that of the DAPI channel (blue) denoting the nuclei. (JPG 220 kb)

Additional file 2: **Figure S2.** Statistic analysis of the shifts of the stripes of Even-skipped (Eve) and Empty spiracles (Ems) in *trans*-Golgin mutants. i: RNAi. ※ 0.05 < *P* < 0.01; ※※ *P* < 0.01. The error bars indicate standard deviation. (JPG 1101 kb)

## Data Availability

The datasets generated during and/or analyzed during the current study are available from the corresponding author on reasonable request.

## Abbreviations

aMTOC: Acentriolar microtubule organizing center
ARTS: Active mRNA transport, synthesis
Bcd: Bicoid
Ems: Empty spiracles
Eve: Even-skipped
MT: Microtubule
SDD: Synthesis, diffusion, degradation
