# Supplementary figures and images for: bicoid RNA localization requires the trans-Golgi network
Source: Hereditas. 2019 Sep 10;156:30. doi: 10.1186/s41065-019-0106-8 (PMC6737670; doi:10.1186/s41065-019-0106-8)

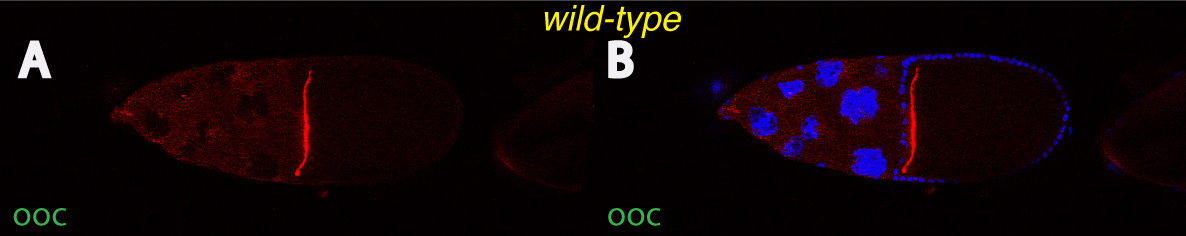

Supplement: Supplementary file 2 — Figure S1. bcd mRNA expression in wild-type embryos. bcd expression pattern in wild-type oocytes, as assayed by fluorescent in situ hybridization. (A) bcd mRNA pattern as a single signal in red, (B) merge of the mRNA signal (red) with that of the DAPI channel (blue) denoting the nuclei. (JPG 220 kb) [file 41065_2019_106_MOESM1_ESM.jpg]

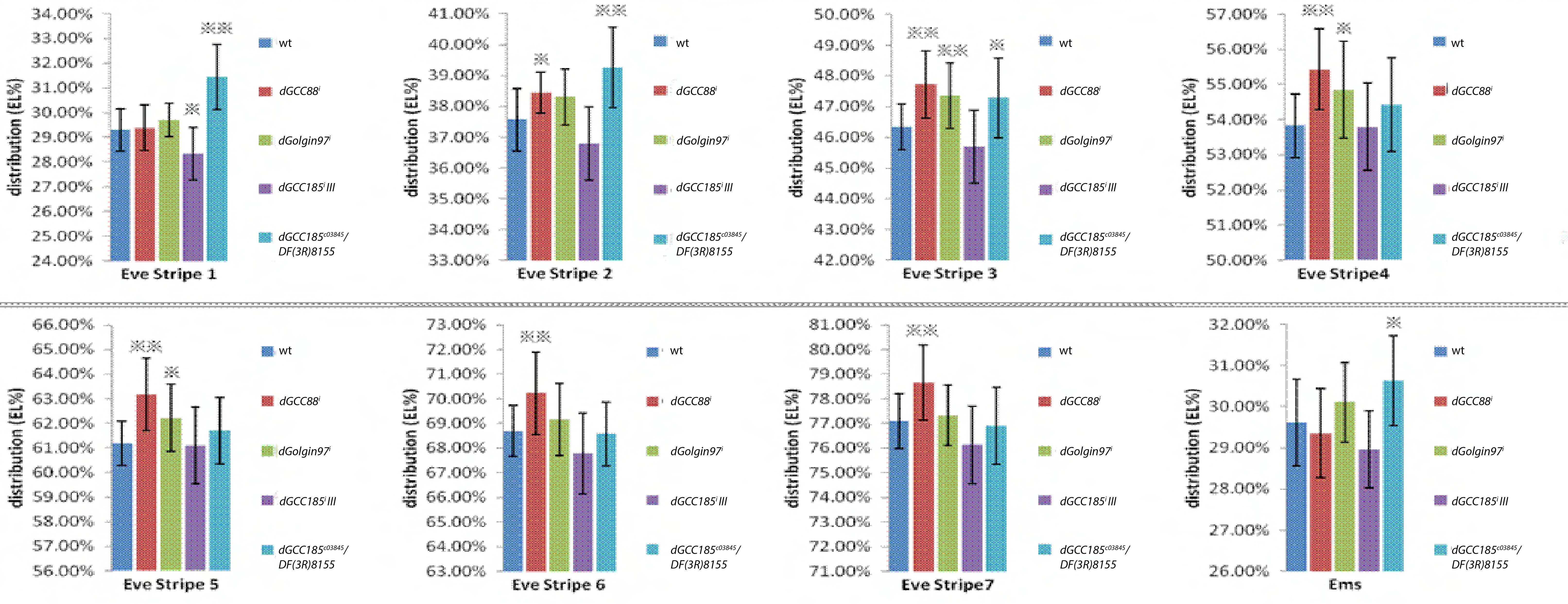

Supplement: Supplementary file 3 — Figure S2. Statistic analysis of the shifts of the stripes of Even-skipped (Eve) and Empty spiracles (Ems) in trans-Golgin mutants. i: RNAi. ※ 0.05 < P < 0.01; ※※ P < 0.01. The error bars indicate standard deviation. (JPG 1101 kb) [file 41065_2019_106_MOESM2_ESM.jpg]
